# Multiple drug resistance caused by germline mutation of exon 27 of BRCA2 gene in triple-negative breast cancer: a case report and literature review

**DOI:** 10.3389/fonc.2025.1602870

**Published:** 2025-06-19

**Authors:** Yuting Li, Guojie Xu, Liling Zhang, Kewei Zhao, Yanxia Zhao, Dan Han

**Affiliations:** Cancer Center, Union Hospital, Tongji Medical College, Huazhong University of Science and Technology, Wuhan, China

**Keywords:** triple-negative breast cancer, BRCA2, frameshift mutation, treatment resistance, cancer progression

## Abstract

BRCA genes, including BRCA1 and BRCA2, are tumor suppressor genes that play a crucial role in the HRR pathway for double-strand DNA breaks. Mutations in these genes lead to the loss of function of their respective proteins, resulting in HRD and the development of hereditary breast cancer. The BRCA2 gene is located on chromosome 13 at the 13q12.3 region and spans 84kb with 27 exons. Frame shift mutations are the most common pathogenic genetic alterations observed in BRCA2, particularly within exons 3, 7, 10, 11, 17, 18, 23 and notably exon 11. Breast cancer patients carrying BRCA1/2 mutations are typically responsive to platinum-based chemotherapy as well as radiation therapy and PARP inhibitors due to their impaired HRR capacity. In this case, a sporadic frameshift mutation was identified in exon 27 of the BRCA2 gene, which has not been previously reported. Studies indicate that mutations occurring within exon 27 disrupt the binding between BRCA2 and RAD51 C-terminal domain resulting in embryonic damage and significantly reduced lifespan based on mouse models of breast cancer. Notably, the patient’s mother and grandmother harbor pathogenic point mutations in BRCA2 on chromosome 13, specifically c.10255dup p. Ter3419LeufsTer19. The patient, a young TNBC, exhibited distinct genetic pathogenic features and changes in the BRCA mutation site. Despite undergoing treatment, the patient experienced rapid recurrence and demonstrated resistance to chemotherapy, PARP inhibitors, and immunotherapy, while remaining sensitive to radiotherapy. This case may serve as a valuable reference for diagnosing and treating breast cancer associated with BRCA2 exon 27 mutations.

## Introduction

The BRCA gene, encompassing BRCA1 and BRCA2, is a crucial tumor suppressor gene encoding key functional proteins involved in the DNA homologous recombination process. It serves as a core gene for maintaining human genome stability (1). Homologous recombination repair (HRR) is the primary pathway for repairing double-strand DNA breaks. Germline mutations in BRCA1/2 (gBRCA1/2) genes result in significant loss of function of their respective proteins, leading to deficiency in homologous recombination repair (HRD), increased genetic instability, and subsequent development of malignant tumors (2). Numerous pathogenic germline mutations have been identified in the BRCA gene, closely associated with various cancers, particularly hereditary breast and ovarian cancer as well as pancreatic cancer, prostate cancer, and melanoma. Breast and ovarian cancers account for 5%-10% and 10%-15% of cases respectively (3). International and domestic guidelines recommend testing for BRCA1/2 genes in breast cancer patients aged 35–40 years old. High-throughput sequencing (NGS) has proven to be the most effective method for detecting BRCA exons and adjacent regions (4). Located on chromosome 13 at region 13q12.3, the BRCA2 gene spans approximately 84kb with 27 exons (5). Mutations observed in the BRCA2 gene primarily include point mutations, insertion mutations, and frameshift mutations. Among these types of mutations, frameshift mutation is the most common. A frameshift mutation refers to a type of DNA mutation where deletion, insertion nonsense mutations of a base at specific points alters the reading frame of a gene resulting in subsequent codon changes that transform its original peptide chain into an entirely different peptide sequence (truncated protein) (6). The most frequently observed pathogenic mutation sites of BRCA2 are exons 3, 7, 10, 11, 17, 18, and 23, with particular emphasis on exon 11 (7). Patients harboring BRCA1/2 mutations are known to exhibit sensitivity towards platinum-based chemotherapy, radiation therapy, and Poly ADP Ribose Polymerase (PARP) inhibitors due to defects in HRR mechanisms (8, 9). In this case, the patient’s mother and grandmother carried pathogenic point mutations in chromosome 13 of BRCA2 at position c.10255dup p. Ter3419LeufsTer19. The patient possessed a pathogenic frameshift mutation in exon 27 of BRCA2 at position c.10255dup p.*3419Leuext*18, and the familial genetic disease predisposition is apparent. Notably, this frameshift mutation within exon 27 of the patient’s BRCA2 gene represents an exceedingly rare occurrence that has not been reported previously. Previous studies have indicated that exon 27 serves as the binding site for BRCA2-RAD51 C-terminal interaction and that mutations within this region lead to embryonic damage and a significant reduction in lifespan in mouse models of breast cancer (10).

This paper presents a unique case of young triple-negative breast cancer (TNBC) patient who experienced rapid recurrence and exhibited primary resistance towards chemotherapy, PARP inhibitors, and immunotherapy, but was sensitive to SBRT stereotactic body radiation therapy (SBRT). This case may provide valuable insights in the diagnosis and treatment of breast cancer patients with frameshift mutation in exon 27 of BRCA2.

## Case presentation

**Figure 1:**
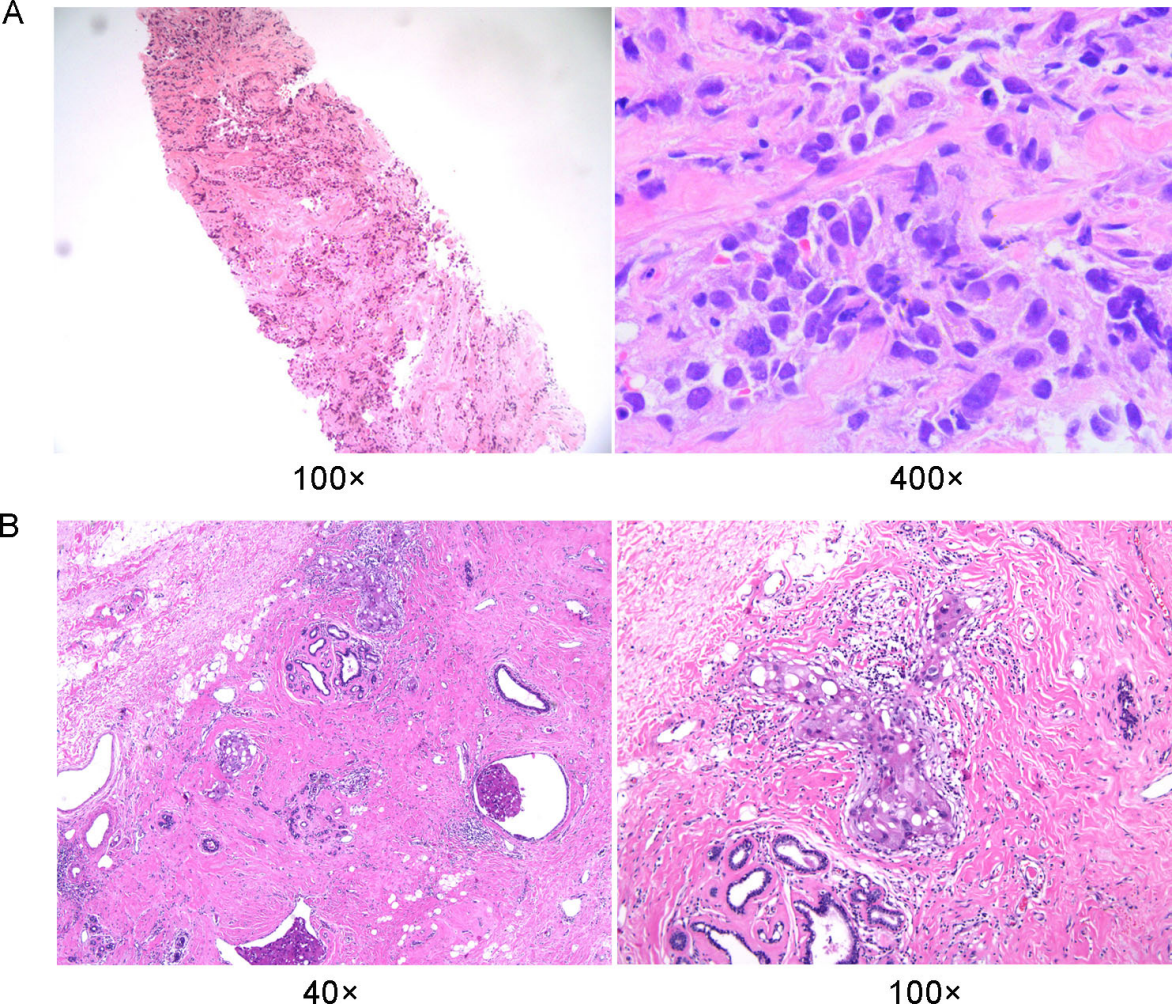
Histopathological findings of breast tumor foci. **(A)** Histopathology of preoperative puncture biopsy of the lesion. **(B)** Histopathology of definitive surgical specimen removal.

**Figure 2:**
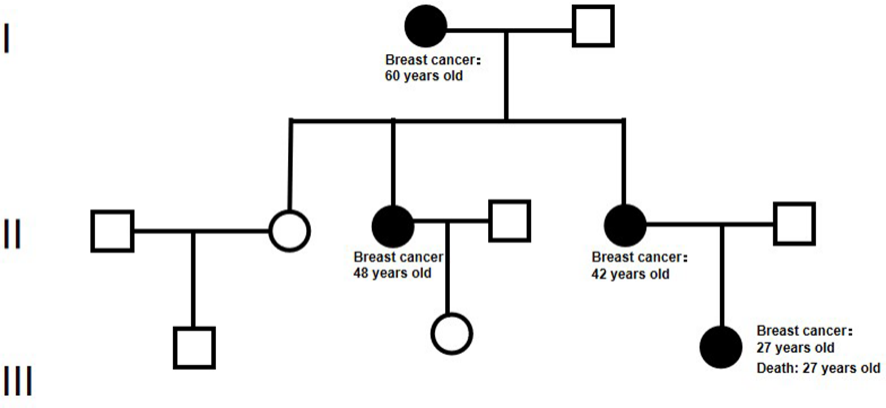
The pedigree diagram of BRCA 2 mutation in a Chinese family. In this chart, squares denote male individuals while circles signify female individuals. Members diagnosed with breast cancer and possessing the gene mutation are marked by filled black circles.

A 27-year-old Chinese woman presented to the local hospital on October 14, 2021, with a self-detected right breast mass persisting for over six months. Enhanced CT scan revealed: 1. A malignant lesion measuring 7.2 cm in diameter and 2.0 cm in thickness in the right breast, 2. Enlarged lymph nodes in the right axillary regions where the largest lymph node measured 1.1 cm in short diameter. Subsequent ^18^F-FDG PET/CT scan confirmed a soft tissue mass in the right breast with abnormally increased glucose metabolism, consistent with the right supraclavicular region, right axillary region, and right lymph node metastasis of the sternum. On October 15, 2021, a core needle biopsy was performed at our hospital which histopathological diagnosed invasive ductal carcinoma (histological grade: III) (**Figure 1A**). Immunohistochemical staining showed: CK7 (+), TRPS 1 (+), BER-EP4 (-), Calretinin (-), WT 1 (-), ER (-), PR (-), AR (-), HER 2 (0), FOXC 1 (partial +), CD8 (-), DCLK 1 (-), Ki67 (LI: 20%). According to the immunohistochemical typing of TNBC by the Cancer Hospital of Fudan University in Shanghai, the test results were classified into immunosuppressive subtypes, and the PD-L1 Combined Positive Score (CPS) was less than 1. The patient underwent 8 cycles of EC (liposomal doxorubicin+cyclophosphamide)-T (albumin doxorubicin) chemotherapy from October 26, 2021, to March 25, 2022, resulting in a partial response (PR). On April 13, 2022, the patient underwent mastectomy with breast reconstruction and skin flap repair on the right side at our hospital’s Breast and Thyroid Surgery Department. Postoperative pathological analysis revealed limited presence of high-grade ductal carcinoma *in situ* without invasive carcinoma tissues. Based on the new adjuvant chemotherapy pathological MP assessment system, the patient’s response was classified as G5 (**Figure 1B**). No tumor involvement was observed at surgical margins or within the breast tissue. However, cancer cells were detected in 18 out of 35 lymph nodes in the right axillary region. On May 24, 2022, the patient underwent radiotherapy in the right supraclavicular lymph node area+right chest wall+right internal breast lymph node area (CTV 50Gy/25f), and an enhanced plan of CTV1 10Gy/5f in the right supraclavicular lymph node area. Genetic testing revealed a frameshift mutation in the 27th exon of BRCA2, specifically c.10255dup p.*3419Leuext*18 with a frequency of 36.85%. Her mother, who also has a history of breast cancer, carries a point mutation on the 13th chromosome of BRCA2 (c.10255dup p. Ter3419LeufsTer19) with a frequency of 42.22%. Similarly, her grandmother without breast cancer has the same point mutation on the 13th chromosome of BRCA2 (c.10255dup p. Ter3419LeufsTer19) with a frequency of 40.46% (**Figure 2**). Considering these findings, our team determined that the patient had a high genetic susceptibility and initiated PARP inhibitor therapy in July 2022.

**Figure 3:**
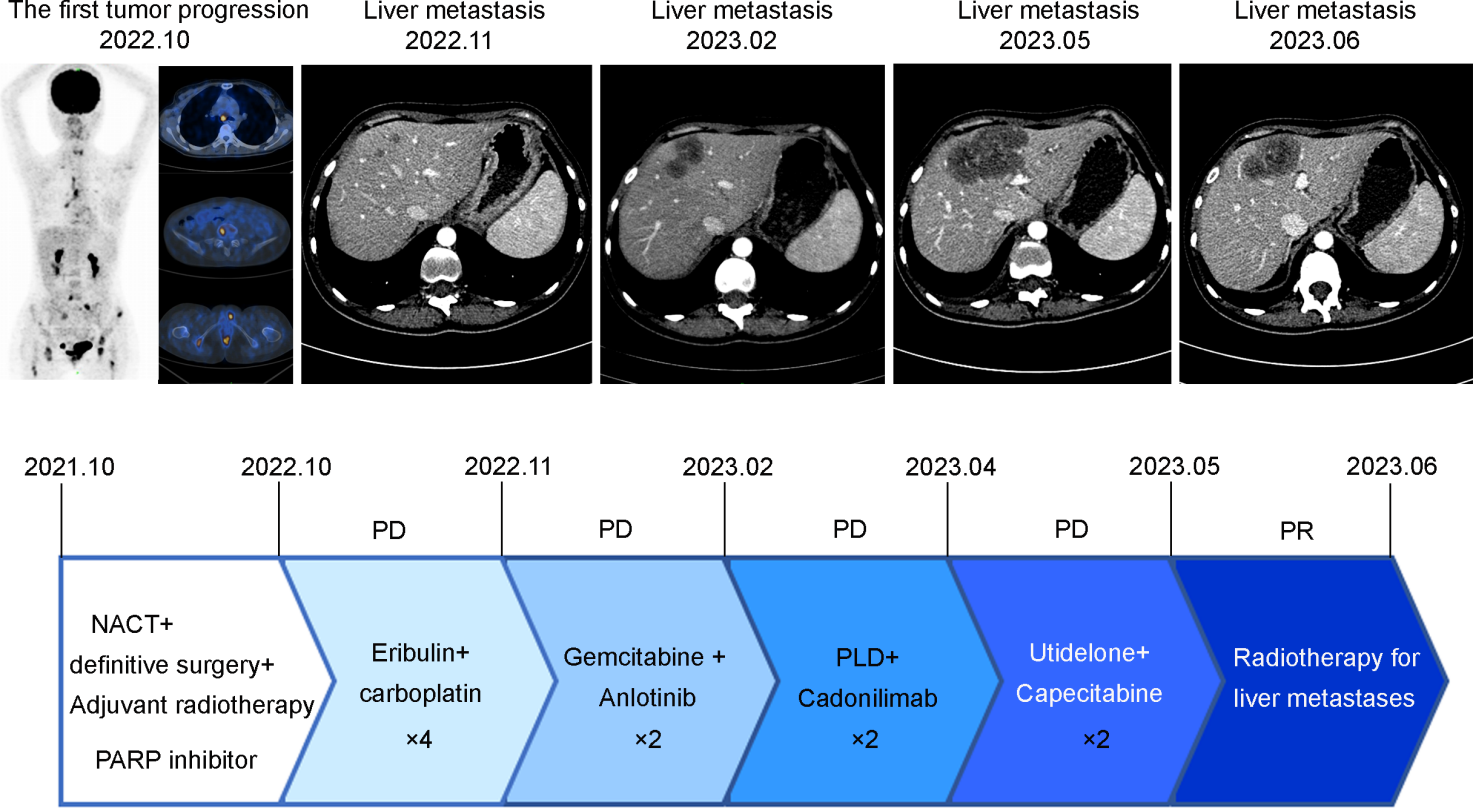
Representative imaging and treatment flow charts of the patient at each stage.

During a follow-up MRI conducted at a local hospital in October 2022 due to left coccygeal pain, multiple abnormal signals in the pelvis and femoral heads indicating metastasis. Furthermore, the ^18^F-FDG PET/CT scan performed on September 29, 2022, revealed: 1. Metabolic abnormalities suggestive of multiple bone metastases were detected in the right clavicle, left humerus, sternum, various vertebrae as well as both femoral heads, 2. Multiple enlarged lymph nodes in the mediastinal regions 1R, 2R, 4R, and 7, with abnormal metabolic increase, were considered for lymph node metastasis. From October 7 to November 7, 2022, the patient underwent four cycles of chemotherapy with Eribulin and carboplatin. On November 20, 2022, an enhanced CT scan revealed multiple nodular enhanced shadows in the right lobe of the liver, suggesting possible metastatic tumors with the largest measuring 1.1*1.0cm. This finding indicated disease progression due to new liver metastatic lesions. Between January 7 and January 29, 2023, two cycles of gemcitabine and Anlotinib (a tyrosine kinase inhibitor) were administered. On February 20, 2023, another enhanced CT scan showed an increase in the size of the liver lesion to 3.8*2.5cm. The patient exhibited a low PD-L1 combined positive score (CPS) and an immunosuppressive immune phenotype. Despite being informed by family members about the limited efficacy of PD-1 immune checkpoint inhibitors, the patient nonetheless requested their use. Consequently, from February 21 to March 15, 2023, the patient received two cycles of liposomal doxorubicin and Cadonilimab (a PD-1/CTLA4 immune checkpoint inhibitor). A subsequent enhanced CT scan on April 4, 2023 demonstrated further enlargement of the liver lesion, which measured at 5.8*3.1cm. On April 7, 2023, the patient underwent two cycles of Utidelone combined with capecitabine treatment. On May 18, 2023, an enhanced CT scan revealed an increase in the size of the liver metastasis lesion to 7.1*5.0 cm. The patient experienced significant upper abdominal pain and subsequently initiated SBRT for the liver metastasis PTV at a dose of 35Gy/5f on May 31. As a result, the patient’s upper abdominal pain was significantly alleviated. On June 27, 2023, an enhanced CT scan demonstrated new findings including pulmonary edema and a substantial increase in right pleural effusion compared to the previous examination, however, there was a reduction in the size of multiple liver metastasis lesions to 5.8*4.1 cm compared to prior assessment. Unfortunately, due to a worsening lung infection, the patient discontinued further treatment later on. Representative imaging and treatment flow charts of the patient at each stage are shown in **Figure 3**.

## Discussion

**Figure 4:**
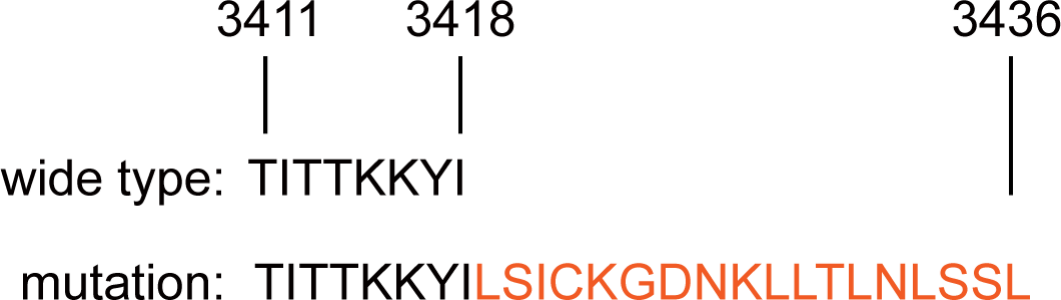
The comparation of the amino acid sequences of wild-type and newly mutated BRCA2 proteins.

Currently, there is a contentious debate surrounding the impact of BRCA2 mutations on overall survival in breast cancer patients (11). BRCA2 is conventionally believed to form a complex with RAD51, facilitating homologous recombination DNA repair. RAD51, as a core recombinase, binds to single-stranded DNA generated during double-strand DNA breaks, promoting not only the repair of these breaks but also safeguarding newly synthesized DNA from degradation during replication (12, 13). Within BRCA2, two regions are capable of binding RAD51: a central octapeptide BRC repeat sequence and a C-terminal RAD51 binding site (14). The exon27 of BRCA2 is presumed to be the location for the BRCA2-RAD51 C-terminal binding site. Greg Donoho et al. demonstrated that alterations in this binding site result in severe embryonic damage and reduced lifespans in mice with 27 exon mutations (10). In this case, the mutation of exon 27 of BRCA2 made the 18 amino acids extension in C-terminal binding site, suppressing BRCA2 binding to RAD51 and breast cancer progression (**Figure 4**). The patient exhibited an elevated risk of breast cancer and a worse prognosis compared to her grandmother and mother.

PARP is a crucial DNA repair enzyme that plays a pivotal role in maintaining genome stability and repairing DNA damage. PARP inhibitors, a novel class of targeted drugs exploiting synthetic lethality, specifically target cancer cells with genetic defects in DNA damage repair and revolutionize the treatment of gBRCA1/2 breast cancers (15). The Olympia study represents the first multicenter, phase III randomized controlled clinical trial investigating the application of PARP inhibitors in primary surgically resectable breast cancer treatment. A total of 1836 high-risk patients with gBRCA1/2 and HER2-negative primary breast cancer were enrolled in this study. Interim analysis results demonstrate significantly improved progression-free survival and overall survival outcomes for the Olaparib group compared to the placebo group (16). Based on these clinical trial findings, both US National Comprehensive Cancer Network (NCCN) guidelines and Chinese Society of Clinical Oncology (CSCO) guidelines recommend Olaparib as an adjuvant therapy option for high-risk early-stage breast cancer patients with BRCA1/2 germline mutations and HER2 negativity (17). Combining PARP inhibitors with platinum-based agents is believed to enhance therapeutic benefits for patients with BRCA-mutated breast cancer. Studies have shown that carboplatin exhibits superior efficacy compared to docetaxel in TNBC patients harboring germline BRCA mutations, achieving an overall response rate (ORR) that exceeds a one-fold increase (18). The BROCADE3 phase III clinical trial indicates that adding veliparib to paclitaxel and carboplatin regimen prolongs progression-free survival (PFS) by 1.9 months among advanced breast cancer patients carrying BRCA mutations (19). Despite the benefits conferred by PARP inhibitors in patients with gBRCA1/2 breast cancer, sustaining these advantages remains challenging. Known mechanisms of PARP inhibitor resistance encompass HRR activity restoration, PARP sequestration of damaged DNA, replication fork stabilization, increased drug efflux, and overactivation of alternative pathways (15). Currently, no effective strategies exist to overcome these mechanisms. The patient exhibits primary resistance to both PARP inhibitors and chemotherapy involving platinum agents; however, the underlying mechanism is unknown. Notably, the BRCA2 27th exon mutation site may serve as a primary resistance determinant for PARP inhibitors and platinum agents, warranting further investigation.

Currently, there is a lack of definitive study results supporting the potential benefits of immunotherapy for breast cancer patients with BRCA1/2 mutations. Some studies have reported higher levels of tumor-infiltrating lymphocytes and tumor mutation burden in BRCA mutation TNBC compared to non-mutated patients, along with elevated expression levels of key immune-related genes PD-1 and CTLA-4 (20). However, other studies have shown no significant differences in tumor-infiltrating lymphocyte score, PD-1, and PD-L1 expression levels between BRCA-mutated TNBC patients and non-mutated patients (21). The TOPACIO Phase II clinical trial demonstrated an ORR of 21% for niraparib + pembrolizumab treatment in treatment-naive metastatic TNBC patients, this ORR was higher among BRCA1/2 mutation carriers and those with PD-L1 positive tumors (47% and 32%, respectively) (22). In the MEDIOLA study evaluating Olaparib combined with durvalumab efficacy in advanced breast cancer patients with germline BRCA mutations, a disease control rate (DCR) at 3 months was observed as 80%, while the ORR reached 63.3% (23). However, the first randomized study combining immunotherapy with PARP inhibitors (atezolizumab + Olaparib) for germline BRCA1/2 mutated advanced breast cancer did not demonstrate significant PFS benefit (15). The patient was classified as an immune-suppressive subtype according to Fudan University’s TNBC classification system, characterized by a low PD-L1 combined positive score (CPS) of less than 1, indicating limited potential benefit from immunotherapy. Consequently, rapid disease progression occurred following the administration of chemotherapy combined with Cadonilimab, a PD-1/CTLA-4 immune checkpoint inhibitor.

The relationship between BRCA2 mutations and the response to stereotactic body radiation therapy (SBRT) in breast cancer reveals a complex interplay characterized by enhanced tumor radiosensitivity. This understanding arises from the critical role of BRCA2 in homologous recombination repair (HRR). When BRCA2 is defective, it renders cells particularly susceptible to DNA double-strand breaks (DSBs) induced by ionizing radiation (24, 25). However, individuals with BRCA-mutated breast cancer also possess an allele that retains partial DNA repair capability, making them susceptible to acute or chronic radiation damage (26). Efimova et al. discovered that combining PARP inhibitors with radiation therapy can enhance the lethal impact of radiation on BRCA1/2-deficient breast cancer cells and expedite cellular senescence (27). It is worth noting that radiation therapy may lead to immune suppression, which can be reversed through combination immunotherapy (28). Following liver SBRT, the patient experienced rapid alleviation of abdominal pain and a significant reduction in the size of the liver metastatic lesion compared to its previous state.

In this case, the differential sensitivity of BRCA2-mutated breast cancer to chemotherapy, immunotherapy, and PARP inhibitors as opposed to radiotherapy arises from the unique role of BRCA2 in DNA damage repair and its broader implications for tumor biology and treatment resistance. BRCA2 exon 27 mutant cancer cells exposed to cisplatin or PARP inhibitors facilitated secondary genetic changes in the mutant alleles, thereby restoring protein function and conferring resistance to chemotherapy and PARP drugs. The PD-L1 combined positive score (CPS) of less than 1, indicating limited potential benefit from immunotherapy. Although these tumors frequently demonstrate intrinsic or acquired resistance to systemic therapies, their dependence on error-prone DNA repair mechanisms makes them particularly vulnerable to ionizing radiation.

## Conclusions

This study reports on a young TNBC woman with an extremely rare frameshift mutation in exon 27 of the BRCA2 gene, which has not been reported in any previous case. Both the patient’s mother and grandmother carried the pathogenic point mutation in BRCA2 on chromosome 13, specifically c.10255dup p. Ter3419LeufsTer19. The patient was resistant to PARP inhibitors, chemotherapy, and immunotherapy and relapsed rapidly, but was sensitive to radiotherapy. This case may provide a valuable reference for the diagnosis and treatment of breast cancer patients with mutations in the BRCA2 gene exon 27.

## Data Availability

The raw data supporting the conclusions of this article will be made available by the authors, without undue reservation.
